# Adenocarcinoma of the esophagogastric junction and its background mucosal pathology: A comparative analysis according to Siewert classification in a Japanese cohort

**DOI:** 10.1002/cam4.1763

**Published:** 2018-09-21

**Authors:** Masayuki Urabe, Tetsuo Ushiku, Aya Shinozaki‐Ushiku, Akiko Iwasaki, Sho Yamazawa, Hiroharu Yamashita, Yasuyuki Seto, Masashi Fukayama

**Affiliations:** ^1^ Department of Gastrointestinal Surgery Graduate School of Medicine The University of Tokyo Tokyo Japan; ^2^ Department of Pathology Graduate School of Medicine The University of Tokyo Tokyo Japan

**Keywords:** adenocarcinoma, Barrett's cancer, esophagogastric junction, gastric cancer, Siewert classification

## Abstract

Adenocarcinoma of the esophagogastric junction (AEG) has heterogeneous carcinogenic process due to its location straddling the esophagogastric junction. We assessed background mucosal pathology and its correlation with clinicopathological features of each Siewert type of AEG. Clinicopathological and immunohistochemical analyses of 103 AEGs and 58 gastric cancers (GCs) were conducted. Background mucosal features were evaluated according to the updated Sydney System. Siewert classification divided 103 AEGs into three type I, 75 type II, and 25 type III tumors, respectively. Two type I, 9 type II AEGs, and none of type III AEGs were Barrett‐related and were excluded from further analysis. Background mucosa of type III AEGs more frequently showed moderate to marked degree of atrophy and intestinal metaplasia than those of type II AEGs and was very similar to those of GCs. Among type II AEGs, tumors with atrophic background were significantly associated with higher patient age and intestinal‐type histology. Type II AEGs with nonatrophic background, but not those with atrophic background, showed more frequent mismatch repair deficiency, TP53 overexpression, and less frequent intestinal phenotypic markers expression than type III AEG or GC. Type II AEGs with atrophic background involved suprapancreatic nodes more frequently than those without. We demonstrated that chronic atrophic gastritis was a major precancerous condition of AEG in the Japanese population, especially Siewert type III which had background mucosal pathology similar to that of GC. Type II AEGs with and without atrophic background showed some clinicopathological differences, and these observations might represent heterogeneous carcinogenic process within type II AEGs.

## 1 INTRODUCTION

The incidence of adenocarcinoma of the esophagogastric junction (AEG) has markedly increased in Western countries during the last few decades.^1^ The risk factors for AEG essentially parallel those of Barrett's adenocarcinoma in these countries. Gastroesophageal reflux disease (GERD), as well as increasing body weight and obesity, is strongly associated with an increased risk of AEG.^2^ On the other hand, studies have reported a reduced risk of Barrett's esophagus and esophageal adenocarcinoma among individuals who are positive for *Helicobacter pylori* gastritis.^2^ In a population like Japanese with a high prevalence of chronic atrophic gastritis as a result of *H. pylori* infection, background mucosal condition and the carcinogenic process of AEG are thought to differ from those of patients in Western countries and instead rather closely resemble those of patients with gastric cancer (GC). However, pathological features including the background mucosal pathology of AEG, and how it compares with those of GC, have yet to be fully elucidated.

Owing to its unique location straddling the junction of the esophagus and stomach, AEG is assumed to be a heterogeneous tumor entity originating from different mucosal types and conditions. These include Barrett's esophageal adenocarcinoma which is associated with GERD and obesity, and gastric adenocarcinoma which is most often associated with *H. pylori* gastritis. According to the Siewert classification, an anatomical subclassification system for AEG,^3^ type I AEG (ie epicenter of which locates 1‐5 cm above the esophagogastric junction [EGJ]) represents adenocarcinoma arising from Barrett's esophagus.^2^, ^4^, ^5^, ^6^ On the other hand, type III AEG (the epicenter is located 2‐5 cm below the EGJ) mainly includes adenocarcinoma arising from the gastric subcardia and is postulated to undergo the same carcinogenic processes as GC in general.^7^, ^8^ Type II AEG ranges in locations from 1 cm above to 2 cm below the EGJ and almost equally involves the two organs. Therefore, its oncogenic background is a matter of considerable debate^4^, ^9^, ^10^ and determining the optimal therapeutic strategy for this entity is also highly controversial.^11^, ^12^ In Japan, EGJ carcinoma has been defined as a malignancy (regardless of histological type) with its center located within 2 cm proximal or distal to the EGJ^13^ and is conceptually close to Siewert type II cancer.

This study aimed to clarify the background mucosal condition and its association with clinicopathological features of each Siewert type of AEG in a Japanese cohort, with a particular focus on the most heterogeneous type II AEGs. We histologically analyzed 103 AEG and 58 GC cases as a control for adjacent nonneoplastic mucosa based on the updated Sydney System as well as cancer tissues in detail. Next, we divided type II AEGs into two groups based on the degrees of background mucosal atrophy and compared their clinicopathological features. We also compared these features to those of type III AEGs and GCs. We then performed immunohistochemical analyses of a panel of major GC‐associated molecules (TP53, HER2, ARID1A, and mismatch repair molecules), mucin phenotypic markers (MUC5AC, MUC6, MUC2, CD10, and CDX‐2), and in situ hybridization for Epstein‐Barr virus‐encoded small RNA (EBER‐ISH) for each group. Finally, we tested whether the background mucosal condition has any clinical significance as a biomarker in type II AEGs, such as determining patient prognosis or predicting the pattern of nodal metastasis, which might provide insights aiding the selection of therapeutic strategies.

## 2 MATERIALS AND METHODS

### 2.1 Case selection

This study included 103 consecutive cases of AEG surgically resected between October 2001 and July 2014 at the University of Tokyo Hospital, Tokyo, Japan. AEG was defined as “a cancer with the center located within 5 cm proximal and distal to the anatomical EGJ as well as infiltrating the EGJ” according to the Siewert system.^3^ Patients with AEGs underwent either total gastrectomy (n = 68), proximal gastrectomy (n = 26), or subtotal esophagectomy (n = 9). We also included 58 consecutive cases of GC, surgically resected from January to July 2009, for comparison. Patients who had received preoperative chemotherapy, radiotherapy, or endoscopic resection were excluded from this study. Patients with remnant stomach were also excluded.

All procedures followed were in accordance with the ethical standards of the responsible committee on human experimentation (institutional and national) and with the Helsinki Declaration of 1964 and later versions. This retrospective study was approved by the Ethics Committee of the Faculty of Medicine and Graduate School of Medicine of the University of Tokyo and the University of Tokyo Hospital.

### 2.2 Clinical data

The clinical data were obtained by reviewing the medical records. Tumor staging was performed using the eighth edition of TNM classification system.^14^ Lymph node stations were determined according to the classification system established by the Japanese Gastric Cancer Association.^13^

### 2.3 Histological evaluation

All of the assessments were performed by two observers (M.U. and T.U.) using a multi‐headed microscope. Hematoxylin and eosin‐stained sections were available in all cases and were evaluated for the following histologic features: histologic type according to Lauren's classification, depth of tumor invasion (T‐classification), nodal metastasis (N‐classification), and lymphovascular invasion.

**Figure 1:**
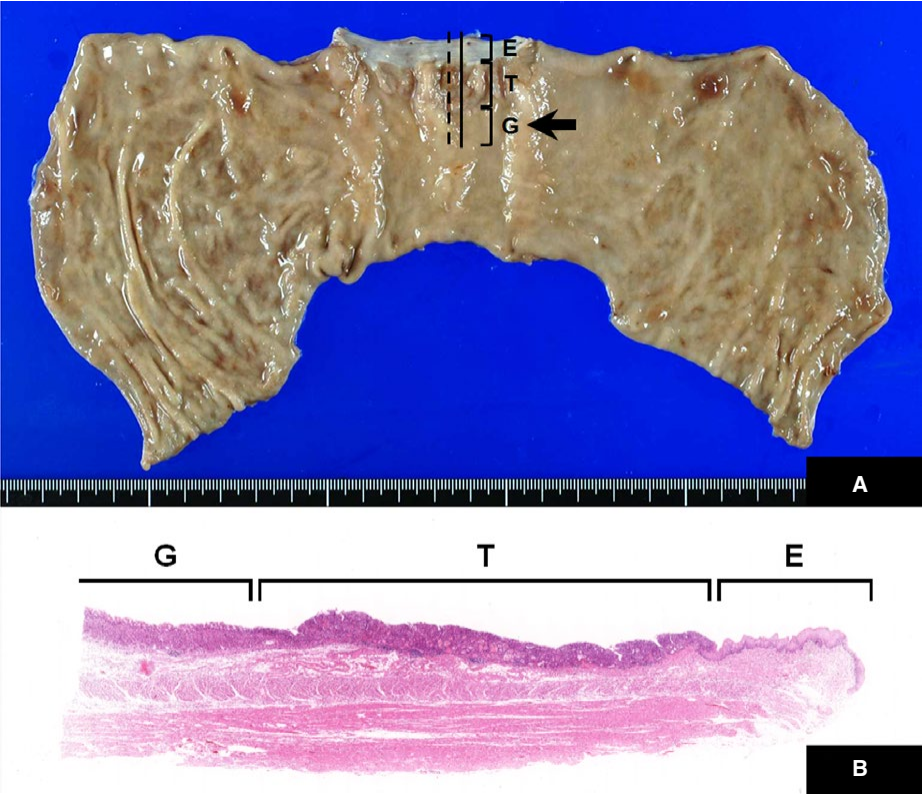
Histologic evaluation of background mucosa adjacent to AEG. A, A 10 mm longitudinal range of glandular mucosa on the lesser curvature immediately adjacent to the anal side of the primary tumor (arrow) was histologically evaluated. E, proximal esophageal squamous epithelium; T, tumor; G, distal gastric epithelium as mucosal background. B, Microscopic appearance of the evaluated area

For evaluation of the background mucosal condition, we microscopically examined a 10 mm longitudinal range of nonneoplastic mucosa adjacent to the distal portions of AEGs and GCs (Figure 1). Because three antral GCs involving the duodenum had no adjacent gastric mucosa on the distal side available for evaluation, we evaluated the proximal side mucosa in these cases. We also assessed the antral mucosa in AEG patients who had undergone total gastrectomy (n = 68).

Background mucosal condition was evaluated for the degree of atrophy, intestinal metaplasia (IM), neutrophils, and mononuclear cells according to the four‐grade scale of the updated Sydney System: normal, mild, moderate, and marked.^15^ On the basis of the grading for atrophy and IM, we classified mucosal conditions into two groups: “atrophic” type when more than a mild degree of atrophy and/or IM was present, otherwise “non‐atrophic” type. We also recorded the predominant type of adjacent mucosa: Barrett's mucosa, cardiac gland mucosa, oxyntic mucosa, pyloric gland mucosa, pseudopyloric gland metaplasia, and IM. Pseudopyloric metaplasia is discerned from true pyloric gland tissue based on the nonantral location as well as lack of G cells. IM was further classified as “complete IM” (small intestinal‐type mucosa composed of absorptive cells with brush borders, goblet cells, and occasionally Paneth cells) or “incomplete IM” (identified by the presence of columnar and goblet cells but lacks Paneth cells and brush borders).^16^, ^17^

### 2.4 Immunohistochemistry and EBER‐ISH

Tissue microarrays were constructed for immunostaining and ISH employing a manual tissue arrayer (Beecher Instruments, Inc., Sun Prairie, WI, USA). We obtained punch biopsies and retrieved duplicate 2‐mm‐in‐diameter tissue cores from each donor block and arrayed them in a recipient array block. Each array block contained 48 tissue cores from 24 tumors. Immunohistochemistry was performed for representative cancer‐associated molecules in GC, including TP53, HER2, ARID1A, and mismatch repair (MMR) proteins (MLH1, PMS2, MSH2, and MSH6)^18^, ^19^, ^20^ as well as for markers of intestinal phenotype (CDX‐2, CD10, and MUC2) and gastric phenotype (MUC5AC and MUC6).^21^ Primary antibody and staining conditions are shown in Table S1. Sections 4 μm in thickness from each tissue microarray block were stained using an automated stainer (Ventana Benchmark; Ventana Medical Systems Inc., Tucson, AZ) along with appropriate positive and negative controls. A sample was defined as being positive for tumor tissue when 10% or more of the neoplastic cells showed staining for HER2 (membranous staining with moderate to strong intensity), CDX‐2 (nuclear staining), CD10 (membranous or cytoplasmic staining), MUC2 (cytoplasmic staining), MUC5AC (cytoplasmic staining), and MUC6 (cytoplasmic staining). Tumors were categorized into gastric or intestinal phenotypes if they were positive for gastric (MUC5AC or MUC6) or intestinal markers (MUC2, CD10 or CDX‐2), respectively.^21^ Preservation or loss of nuclear staining was evaluated for ARID1A, MLH1, MSH2, MSH6, and PMS2. MMR deficiency was defined as a tumor showing complete loss of any of the four MMR proteins (MLH1, MSH2, MSH6, and PMS2). The results of TP53 immunohistochemistry were considered positive when neoplastic cells were diffusely positive (>50%) for TP53 staining. Epstein‐Barr virus positivity was determined by EBER‐ISH with a FITC‐labeled peptide nucleic acid probe (Y5200; Dako, Glostrup, Denmark) and anti‐FITC antibody (polyclonal, dilution 1:25; Thermo Fisher Scientific, Waltham, MA).

### 2.5 Statistical analysis

Clinicopathological data were compared by the chi‐squared test for categorical variables and by Student's *t* test for continuous variables. The survival curves were calculated by the Kaplan‐Meier method. Differences between the curves were analyzed employing the log‐rank test. Overall survival was defined as the time from surgery until death from any cause. For all statistical analyses, values of *P *<* *0.05 (two‐tailed) were considered to indicate a statistically significant difference. Statistical analyses were carried out using JMP Pro version 13.0.0 (SAS Institute, Cary, NC).

## 3 RESULTS

### 3.1 Siewert classification and association with Barrett's esophagus

The Siewert classification was applied to dividing 103 AEGs into type I (n = 3, 3%), type II (n = 75, 73%), and type III (n = 25, 24%). Eleven AEGs, including two out of three (67%) type I and nine out of 75 (12%) type II AEGs, were diagnosed as Barrett‐related cancer. On the other hand, none of the type III AEGs were associated with Barrett's mucosa. The one type I AEG case with no features of Barrett's esophagus was present entirely beneath the normal squamous epithelium, and the lesion was assumed to have arisen from the esophageal gland proper.

### 3.2 Clinicopathological features

Table 1 summarizes the clinicopathological features of our cohort for each Siewert subtype. There was no significant difference in patient age, sex, or histological type between type II and type III AEGs. Type III tumors had higher T‐classifications and more frequent nodal metastases than type II AEGs. GCs had lower T‐classifications, N‐classifications, and less frequent lymphovascular invasion than type II and type III AEGs. As for Lauren's histology, diffuse type was relatively frequent in GCs as compared to type II AEGs. Main locations of 58 GCs were as follows: 9 (16%) in the upper third, 36 (62%) in the middle third, and 13 (22%) in the lower third.

**Table 1:** Clinicopathological features of each Siewert type of AEGs and GCs

Group	All cases	AEG			GC	*P* value Type II vs III	*P* value Type II vs GC	*P* value Type III vs GC
		Type I	Type II	Type III				
# of cases	161	3	75	25	58			
Age (years, mean ± SD)	65.1 ± 13.5	52.7 ± 6.4	64.7 ± 13.7	70.7 ± 11.4	63.9 ± 13.8	0.052*	0.73*	**0.033** *
Sex (Male/Female)	128/33	3/0	61/14	21/4	43/15	0.76†	0.32†	0.33†
Lauren's histology (Intestinal/Mixed/Diffuse)	80/69/12	3/0/0	40/33/2	13/10/2	24/26/8	0.49†	**0.042** †	0.60†
Barrett's cancer (Yes/Not determined)	11/150	2/1	9/66	0/25	0/58	0.069†	**0.006** †	NC
T‐classification (≤T2/≥T3)	82/79	3/0	32/43	4/21	43/15	**0.016** †	**<0.001** †	**<0.001** †
N‐classification (N0/≥N1)	78/83	1/2	33/42	5/20	39/19	**0.032** †	**0.008** †	**<0.001** †
M‐classification (M0/M1)	140/21	3/0	66/9	21/4	50/8	0.61†	0.76†	0.79†
Lymphatic involvement (Negative/Positive)	76/85	2/1	30/45	7/18	37/21	0.28†	**0.007** †	**0.003** †
Venous involvement (Negative/Positive)	51/110	3/0	16/59	4/21	28/30	0.56†	**0.001** †	**0.006** †

AEG, adenocarcinoma of the esophagogastric junction; GC, gastric cancer; NC, not calculated; SD, standard deviation.

*Student's *t* test; ^†^Chi‐squared test

Statistically significant values (*P *<* *0.05) are represented in bold style.

### 3.3 Background mucosal types

**Figure 2:**
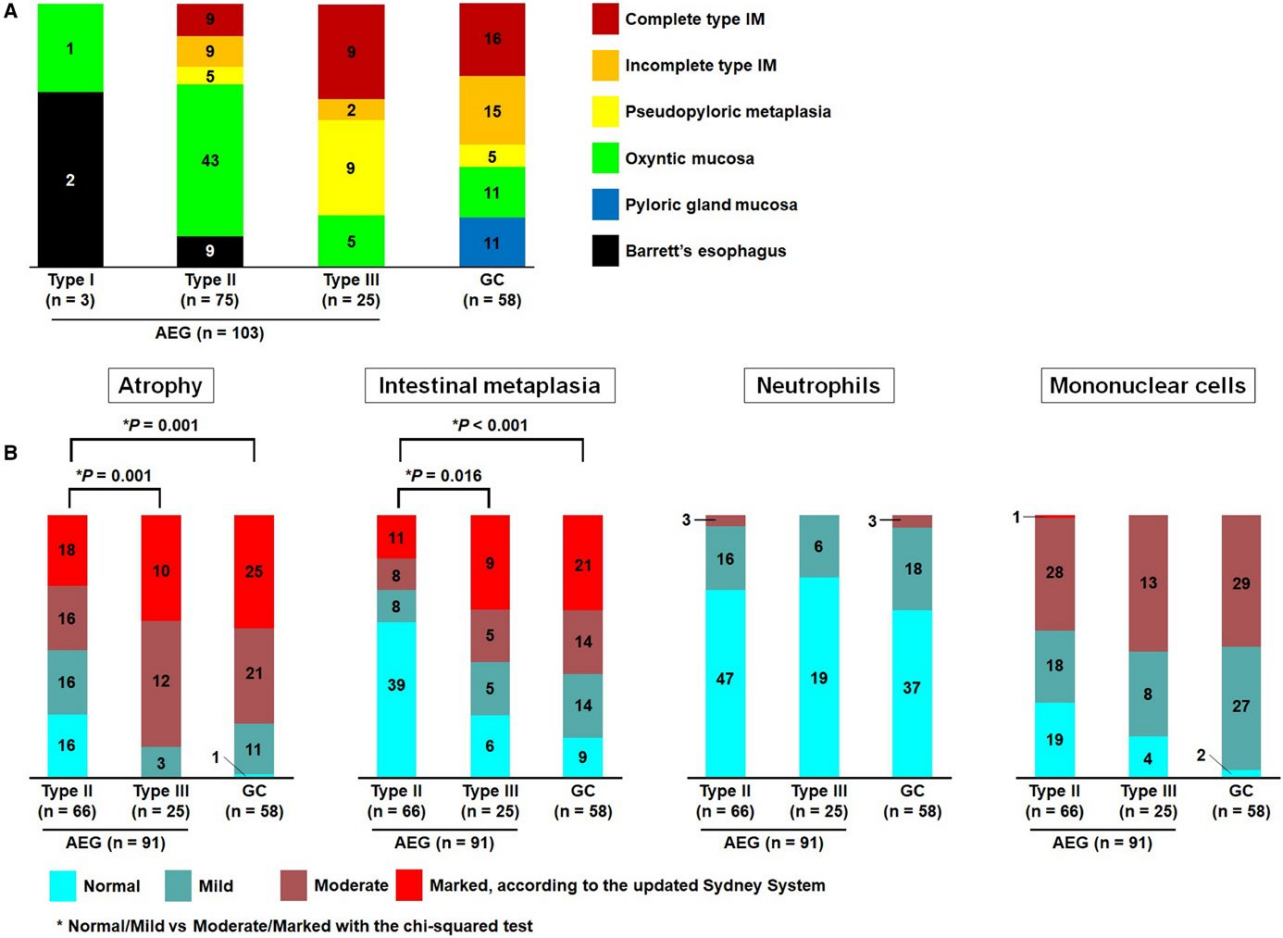
A, Background mucosal type of 103 AEGs and 58 GCs was divided into six categories. B, Background mucosal pathologies of 91 AEGs (excluding 11 Barrett‐related cancers and one cancer associated with the esophageal proper glands) and 58 GCs were evaluated in accordance with the updated Sydney System. Degrees of atrophy, intestinal metaplasia, and neutrophilic and mononuclear cell infiltrations were divided into four grades

Figure 2A summarizes the adjacent mucosal types of 103 AEGs and 58 GCs. Oxyntic mucosa was the most prevalent (n = 43, 57%) among type II AEGs. In contrast, adjacent mucosal features of type III tumors were predominantly metaplastic mucosa (n = 20, 80%) including pseudopyloric metaplasia (n = 9, 36%), complete type IM (n = 9, 36%), and incomplete type IM (n = 2, 4%). Background mucosal types of type III AEGs were more similar to those of GCs than to those of type II AEGs.

### 3.4 Background mucosal pathology according to the updated Sydney System

Background mucosal features of 91 AEGs and 58 GCs were histologically evaluated using the updated Sydney System (Figure 2B). Eleven Barrett‐related AEGs and one AEG associated with the esophageal proper glands were excluded from this analysis.

Moderate to marked atrophy was less frequently observed in the background mucosa of type II AEGs (52%) than in those of type III AEGs (88%, *P *=* *0.001) and GCs (79%, *P *=* *0.001). Similarly, moderate to marked degrees of IM were less frequently noted in the background mucosa of type II AEGs (29%) than in those of type III AEGs (56%, *P *=* *0.016) and GCs (60%, *P *<* *0.001). There was no significant difference in the degree of atrophy and IM between type III AEGs and GCs (*P *=* *0.35, *P *=* *0.71, respectively). The degrees of neutrophil and mononuclear cell infiltration did not differ significantly among the three groups. In total, 17 patients had normal background mucosa with no atrophy or IM, and all but one were considered to have Siewert type II AEGs, the exception being one GC case with signet ring cell carcinoma, whereas the background mucosa of type III AEGs invariably showed at least mild atrophy or IM (Figure 2B). *Helicobacter pylori* colonization was microscopically confirmed in only 35 of 149 cases (18 type II AEGs, 4 type III AEGs, and 13 GCs, *P *=* *0.51). Because *H. pylori* organisms become undetectable in diffuse atrophic metaplastic mucosa, chronic atrophic gastritis in this cohort is highly likely to be a consequence of *H. pylori* infection.^22^ There were no features suggesting other etiology of gastritis, such as autoimmune gastritis, in our cohort.

In AEG patients undergoing total gastrectomy (43 type II and 25 type III), we also assessed the antral mucosa and found that type III AEGs tended to show more atrophy and IM than type II AEGs in the antral mucosa, but the differences did not reach statistical significance (Figure S1).

### 3.5 Relationships between background mucosal condition and clinicopathological features within Siewert type II AEGs

Next, we classified Barrett‐unrelated type II AEGs (n = 66) into two groups: “atrophic” (n = 34) when more than a mild degree of atrophy and/or IM was present, otherwise “non‐atrophic” (n = 32). We compared clinicopathological features among three groups: type II AEGs with an “atrophic” background, those with a “non‐atrophic” background, and type III AEGs plus GCs (combined group; Table 2).

**Table 2:** Comparison of clinicopathological factors among three groups: type II AEGs with an “atrophic” background, those with a “non‐atrophic” background, and combined type III AEGs plus GCs

Group	All cases	Type II AEG		Type III AEG & GC	*P* value		
		Non‐Atrophic (NA)	Atrophic (A)		Type II (NA) vs Type II (A)	Type II (NA) vs Type III & GC	Type II (A) vs Type III & GC
# of cases	149	32	34	83			
Age (years, mean ± SD)	65.7 ± 13.0	60.9 ± 13.5	69.6 ± 10.4	66.0 ± 13.4	**0.004** *	0.072*	0.16*
Sex (Male/Female)	117/32	24/8	29/5	64/19	0.29†	0.81†	0.32†
Lauren's histology (Intestinal/Mixed/Diffuse)	75/62/12	13/19/0	22/10/2	37/36/10	**0.029** †	0.075†	0.13†
T‐classification (≤T2/≥T3)	71/78	10/22	14/20	47/36	0.40†	**0.015** †	0.13†
N‐classification (N0/≥N1)	71/78	12/20	15/19	44/39	0.58†	0.14†	0.38†
M‐classification (M0/M1)	128/21	24/8	33/1	71/12	**0.009** †	0.18†	0.072†
Lymphatic involvement (Negative/Positive)	67/82	9/23	14/20	44/39	0.27†	**0.016** †	0.25†
Venous involvement (Negative/Positive)	44/105	6/26	6/28	32/51	0.91†	**0.043** †	**0.028** †
Immunohistochemistry and EBER‐ISH							
HER2 (Negative/Positive)	101/48	22/10	24/10	55/28	0.87†	0.80†	0.65†
ARID1A (Lost/Preserved)	12/137	2/30	3/31	7/76	0.69†	0.70†	0.95†
MMR proteins (Lost/Preserved)	19/130	9/23	4/30	6/77	0.095†	**0.003** †	0.43†
Gastric phenotype markers (Negative/Positive)	65/84	18/14	14/20	33/50	0.22†	0.11†	0.89†
Intestinal phenotype markers (Negative/Positive)	78/71	22/10	21/13	35/48	0.55†	**0.011** †	0.054†
TP53 (Negative/Positive)	109/40	18/14	25/9	66/17	0.14†	**0.012** †	0.48†
EBER‐ISH (Negative/Positive)	135/14	31/1	31/3	73/10	0.33†	0.14†	0.61†

AEG, adenocarcinoma of the esophagogastric junction; EBER‐ISH, Epstein‐Barr virus‐encoded small RNA in situ hybridization; GC, gastric cancer; MMR, mismatch repair; SD, standard deviation.

*Student's *t* test; ^†^Chi‐squared test

Statistically significant values (*P *<* *0.05) are represented in bold style.

“Atrophic” type II AEG patients were significantly older than those with a “non‐atrophic” background (*P *=* *0.004). Intestinal‐type histology was more frequent in “atrophic” type II AEGs than in the “non‐atrophic” group.

Neither immunohistochemical analyses nor EBER‐ISH revealed any significant differences in markers between “atrophic” and “non‐atrophic” type II AEGs. However, MMR deficiency and TP53 overexpression were more frequent and expressions of intestinal phenotypic markers were less frequent in “non‐atrophic” type II AEGs than in the combined type III AEGs plus GCs group (*P *=* *0.003, 0.011, and 0.012, respectively), although these differences did not reach statistical significance when type III AEGs and GCs were analyzed separately. On the other hand, there were no significant differences in marker expressions between “atrophic” type II AEGs and the combined type III AEGs plus GCs group.

### 3.6 Clinical significance of background mucosal conditions in Siewert type II AEGs

**Figure 3:**
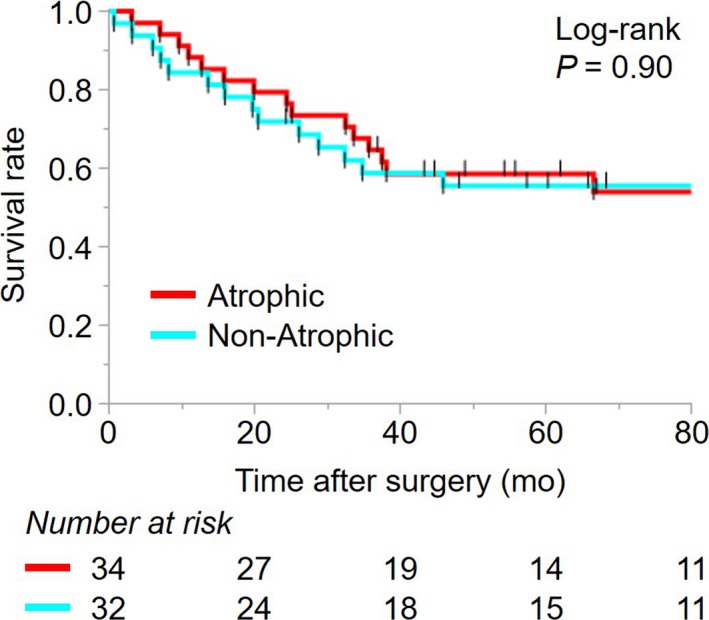
Survival analysis using Kaplan‐Meier methods. Dichotomous comparisons of overall survival among 66 type II AEG patients (excluding those with Barrett‐related cancers) according to grades of atrophy/intestinal metaplasia of the background epithelium (“atrophic” vs “non‐atrophic”)

In total, 66 patients with Barrett‐unrelated type II AEGs were included in this analysis. The median follow‐up period was 48.5 months at the time of the final follow‐up (January 2018). Background mucosal condition (“atrophic” vs “non‐atrophic”) had no significant impact on patients’ overall survivals as determined by Kaplan‐Meier estimation (Figure 3).

**Figure 4:**
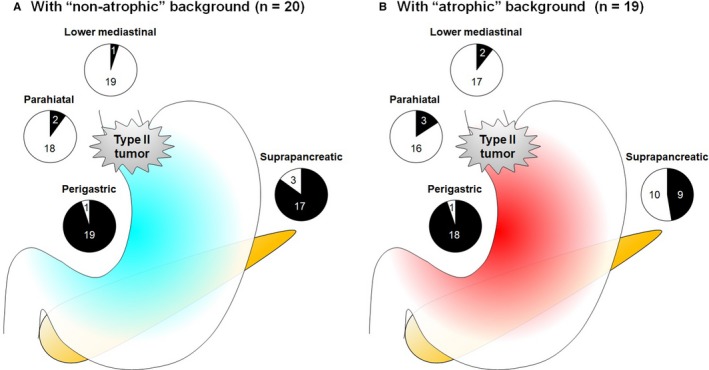
The distribution of lymph node metastases in Siewert type II cancers. A, Node‐positive tumors with a “non‐atrophic” background (n = 20). B, Node‐positive tumors with an “atrophic” background (n = 19). Pie charts for each lymph node station indicate the proportion of node‐positive/negative cases: Black represents node‐positive cases and white node‐negative cases, with numbers of cases. Lymph node stations are defined as follows: “Lower mediastinal” station includes lower thoracic paraesophageal, supradiaphragmatic, and posterior mediastinal nodes; “Parahiatal” station includes infradiaphragmatic nodes and nodes along the esophageal hiatus; “Suprapancreatic” station includes nodes along the left gastric artery, common hepatic artery (anterosuperior side), the celiac artery, splenic hilum, and the splenic artery; “Perigastric” station includes right cardial, left cardial, lesser curvature, suprapyloric, and infrapyloric nodes as well as the nodes along the short gastric artery, the left gastroepiploic artery, and the right gastroepiploic artery

Next, we assessed the sites of lymph node metastases for cases of non‐Barrett's type II AEG with nodal metastasis (n = 39) in the “atrophic” and “non‐atrophic” groups according to the classification system established by the Japanese Gastric Cancer Association (“lower mediastinal”, “parahiatal”, “suprapancreatic,” and “perigastric”; Figure 4). Of note, suprapancreatic nodes were more frequent in type II AEGs with “non‐atrophic” background cases (17 out of 20, 85%) than in those with “atrophic” background cases (nine out of 19, 47%, *P *=* *0.013). Regarding the other nodal stations, there was no significant difference in positive ratios between the two groups. There was no significant difference between the two groups in T‐classification, N‐classification, lymphovascular invasion or main cross‐sectional location (the lesser curvature/the greater curvature/the anterior wall/the posterior wall; Table S2).

## 4 DISCUSSION

Earlier studies evaluated the background mucosal pathologies of AEG.^9^, ^23^, ^24^ They investigated background condition of Barrett's metaplasia and atrophic gastritis caused by *H. pylori* infection in AEG limited to Siewert type II^9^, ^23^ or EGJ cancer in Japanese definition.^13^, ^24^ However, information regarding background mucosal pathologies of AEGs according to the Siewert classification, along with comparison to GC, is still limited. We conducted the first detailed histologic comparison in a Japanese cohort of background mucosal types and conditions, to allow comparisons among each Siewert subtype of AEG, focusing especially on type II AEG, along with comparisons to GC according to the updated Sydney System.

In Western countries, the proportion of type I tumor among AEG is relatively high accounting for 14%‐39% of all AEGs.^25^, ^26^, ^27^ In contrast, previous studies demonstrated that type I AEG is extremely rare (around 1%‐3%) and that type II & III are predominant in Japan,^28^, ^29^ as was confirmed in our study. This geographical difference might be explained by a correlation of GERD and obesity and an inverse correlation of *H. pylori* infection with Barrett‐related adenocarcinoma.^2^, ^4^, ^5^, ^6^ A certain proportion of type II AEGs as well as the majority of type III AEGs are postulated to develop in association with chronic atrophic gastritis caused by *H. pylori,* as is the case with GCs.^8^, ^9^, ^30^ Our study mainly focused on Siewert type II & III AEGs, and we conducted a comparison with GC to reveal the extent of chronic atrophic gastritis involvement in each type of AEG.

We demonstrated that mucosal backgrounds differed significantly between type II and III AEGs. First, Barrett's esophagus was exclusively recognized in the cases with type I & II AEGs and was absent in those with type III AEG. Second, atrophy and IM were significantly less prominent in type II than in type III AEGs. This finding is in line with those of a previous study suggesting that a portion of type II AEGs arises from gastritis‐unrelated mucosa.^9^ In our current study, 16 out of 75 (21%) type II AEGs and none of the type III AEG arose in a background of apparently normal gastric mucosa. Finally, the background mucosal type and condition of type III AEGs are more similar to those of GCs than to those of type II AEGs. This observation supports the notion that type III AEG essentially arises from the upper stomach and shares a carcinogenic process with GC.

In our dichotomous comparison between “atrophic” and “non‐atrophic” groups of type II AEGs, tumors with an “atrophic” background were significantly associated with intestinal‐type histology, features consistent with those described in an earlier publication.^23^ This finding raises the possibility that AEGs with an “atrophic” background develop via the “atrophy‐metaplasia‐carcinoma sequence” which is recognized as a carcinogenic process of intestinal‐type GC.^31^, ^32^ This study indicated that about half of type II AEGs as well as most type III AEGs have an “atrophic” background and may represent this carcinogenic sequence. Our immunohistochemical observations also support this notion because type II AEGs with an “atrophic” background showed immunophenotypes similar to those of the combined type III AEG plus GC group, whereas those with a “non‐atrophic” background more frequently showed MMR deficiency, TP53 overexpression, and negativity for intestinal phenotypic markers, as compared to the combined type III AEGs plus GCs group. This result suggests that type II AEGs with an “atrophic” background are more similar than those with a “non‐atrophic” background to type III AEGs and GCs, in terms of the carcinogenic process.

Although background mucosal pathology did not affect patients’ outcomes in our cohort, it might serve as a biomarker for identifying the heterogeneous nature of type II AEGs. Of note, type II tumors with a “non‐atrophic” background were more likely to be associated with suprapancreatic metastasis than those of the “atrophic” type, which has not previously been reported. Interestingly, Pedrazzani et al^27^ reported that the frequency of suprapancreatic metastasis (left gastric artery, common hepatic artery, celiac trunk, splenic hilum, and splenic artery) was higher in type II than type III AEG. Therefore, in terms of metastatic pattern, type II AEGs with an “atrophic” background are more similar to type III than to type II AEG with a “non‐atrophic” background. In addition, a previous study analyzing a series of adenocarcinomas of the esophagus and cardia demonstrated that tumors without IM in the stomach more frequently involved pancreatic nodes and celiac trunk nodes than tumors with IM (24/43 vs 7/61, *P *<* *0.001; 18/139 vs 3/84, *P *=* *0.02, respectively).^33^ These observations may reflect different biologic behaviors and possibly different carcinogenic pathways within type II AEGs, which could potentially be distinguished by background mucosal condition. Although we must be cautious when interpreting these findings because of the small number of cases analyzed and the absence of multivariate estimation, background mucosal condition may serve as a predictor of the pattern of nodal metastasis in patients with type II AEG.

The limitations of our study include the possibility that overgrowth of AEG might destroy and/or conceal the underlying Barrett's mucosa.^34^, ^35^ This may result in underestimation of the proportion of Barrett‐related cancers among AEGs. The relatively small number of enrolled cases is also an inherent limitation of this study. A larger sample size is necessary to verify the clinicopathologic importance of background mucosal pathology in each subtype.

In summary, we demonstrated chronic atrophic gastritis to be a major precancerous condition of AEG in the Japanese population, especially of Siewert type III which has a background mucosal pathology similar to that of GC. Type II AEGs with an “atrophic” background were more similar to type III AEGs and GCs than to those with a “non‐atrophic” background in terms of both clinicopathological and immunohistochemical features. Therefore, background mucosal pathology might reflect different biologic behaviors and different carcinogenic pathways of heterogeneous type II AEGs.

## CONFLICT OF INTEREST

The authors declare that they have no conflict of interests.

## Supporting information


** **

** **

** **
