# Supplementary figures and images for: Adenocarcinoma of the esophagogastric junction and its background mucosal pathology: A comparative analysis according to Siewert classification in a Japanese cohort
Source: Cancer Med. 2018 Sep 21;7(10):5145–54. doi: 10.1002/cam4.1763 (PMC6198208; doi:10.1002/cam4.1763)

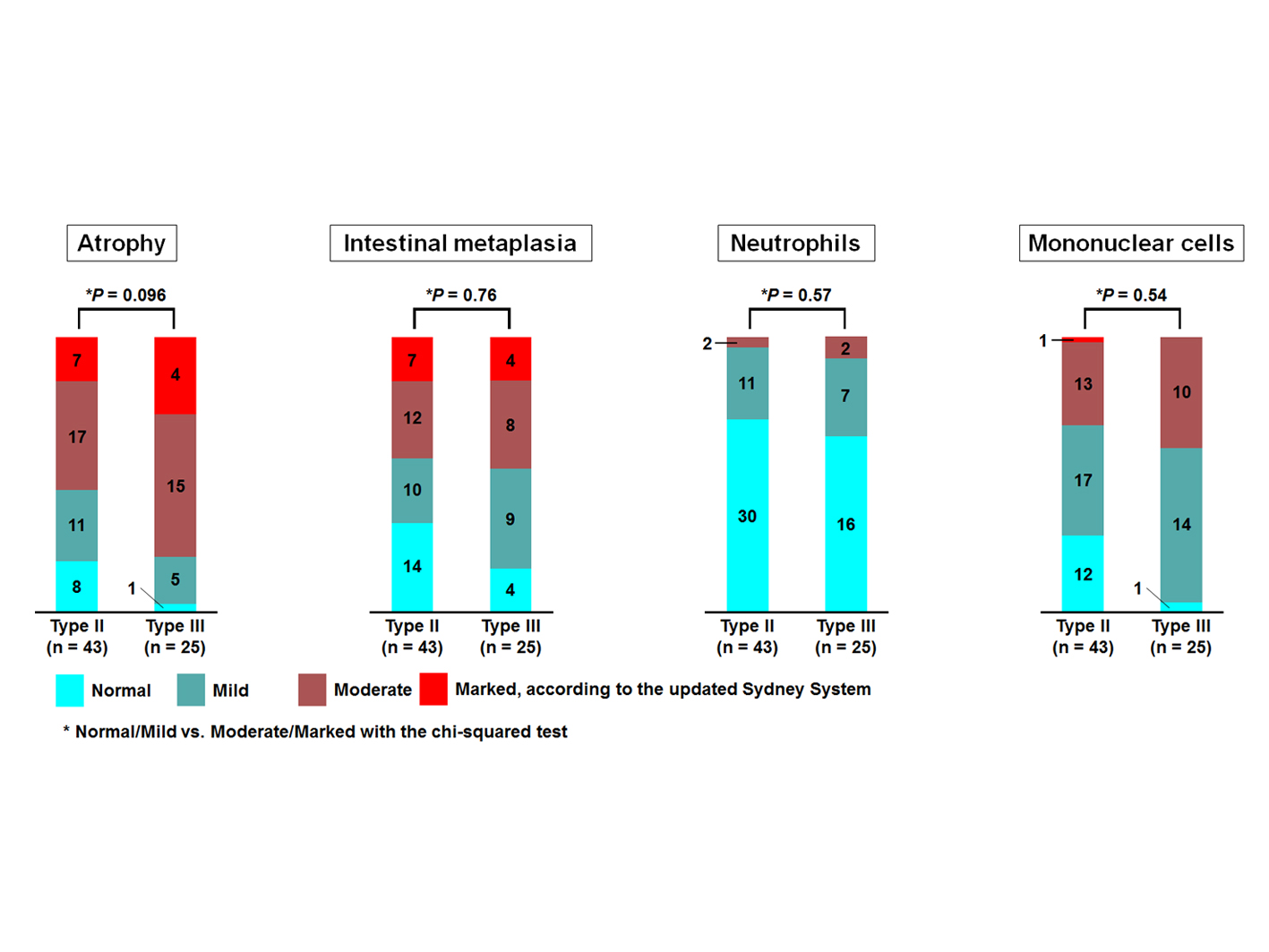

Supplement: Supplementary file 1 [file CAM4-7-5145-s001.tif]
