# Supplementary material for: Adenocarcinoma of the esophagogastric junction and its background mucosal pathology: A comparative analysis according to Siewert classification in a Japanese cohort
Source: Cancer Med. 2018 Sep 21;7(10):5145–54. doi: 10.1002/cam4.1763 (PMC6198208; doi:10.1002/cam4.1763)
Supplement: Supplementary file 2 [file CAM4-7-5145-s002.docx]

| **Supplementary Table S1** Antibodies used for immunohistochemical studies | | | | | |
| --- | --- | --- | --- | --- | --- |
| Antibody | Clone | Type | Source | Dilution | Staining Pattern |
| HER2 | 4B5 | Rabbit, mono | Ventana Medical Systems Inc., Tucson, AZ | Ready to use | M |
| ARID1A | - | Rabbit, poly | Sigma-Aldrich, St. Louis, MO, USA | 1:100 | N |
| MLH1 | ES05 | Mouse, mono | Novocastra–Leica Biosystems, Newcastle Upon Tyne, UK | 1:50 | N |
| MSH2 | FE11 | Mouse, mono | Dako, Glostrup, Denmark | 1:50 | N |
| MSH6 | EPR3945 | Rabbit, mono | GeneTex, LosAngeles, USA | 1:200 | N |
| PMS2 | EPR3947 | Rabbit, mono | Ventana Medical Systems Inc. | Ready to use | N |
| CDX-2 | CDX2-88 | Mouse, mono | BioGenex, San Ramon, CA, USA | 1:200 | N |
| CD10 | 56C6 | Mouse, mono | Novocastra–Leica Biosystems | 1:200 | C, M |
| MUC2 | Ccp58 | Mouse, mono | Novocastra–Leica Biosystems | 1:20 | C |
| MUC5AC | CLH2 | Mouse, mono | Novocastra–Leica Biosystems | 1:100 | C |
| MUC6 | CLH5 | Mouse, mono | Novocastra–Leica Biosystems | 1:100 | C |
| p53 | DO7 | Mouse, mono | Novocastra–Leica Biosystems | 1:50 | N |
| C indicates cytoplasmic; M, membranous; N, nuclear. | | | | | |
