# Supplementary material for: Adenocarcinoma of the esophagogastric junction and its background mucosal pathology: A comparative analysis according to Siewert classification in a Japanese cohort
Source: Cancer Med. 2018 Sep 21;7(10):5145–54. doi: 10.1002/cam4.1763 (PMC6198208; doi:10.1002/cam4.1763)
Supplement: Supplementary file 3 [file CAM4-7-5145-s003.docx]

| **Supplementary Table S2.** Comparison of clinicopathological factors between "non-atrophic" and "atrophic" groups in 39 type II AEGs with nodal metastasis. | | | | |
| --- | --- | --- | --- | --- |
| Group | All cases | Non-Atrophic | Atrophic | *P* value |
| # of cases | 39 | 20 | 19 |  |
| T classification (T1/T2/T3/T4) | 2/2/20/15 | 1/0/12/7 | 1/2/8/8 | 0.42 |
| N classification (N1/N2/N3) | 7/16/16 | 1/10/9 | 6/6/7 | 0.091 |
| Lymphatic involvement (Negative/Positive) | 7/32 | 2/18 | 5/14 | 0.18 |
| Venous involvement (Negative/Positive) | 3/36 | 2/18 | 1/18 | 0.58 |
| Cross-sectional location (Gre/Ant/Less/Post) | 3/5/24/7 | 3/3/10/4 | 0/2/14/3 | 0.26 |
| *Gre* greater curvature, *Ant* anterior wall, *Less* lesser curvature, *Post* posterior wall. | | | | |
